# Early decrease of blood myeloid-derived suppressor cells during checkpoint inhibition is a favorable biomarker in metastatic melanoma

**DOI:** 10.1136/jitc-2023-006802

**Published:** 2023-06-07

**Authors:** Andrea Gaißler, Jonas Bochem, Janine Spreuer, Shannon Ottmann, Alexander Martens, Teresa Amaral, Nikolaus Benjamin Wagner, Manfred Claassen, Friedegund Meier, Patrick Terheyden, Claus Garbe, Thomas Eigentler, Benjamin Weide, Graham Pawelec, Kilian Wistuba-Hamprecht

**Affiliations:** 1 Department of Dermatology, University Hospital Tübingen, Eberhard Karls University of Tübingen, Tübingen, Germany; 2 Internal Medicine I, University Hospital Tübingen, Eberhard Karls University of Tübingen, Tübingen, Germany; 3 Cluster of Excellence iFIT (EXC 2180) “Image Guided and Functionally Instructed Tumor Therapies”, Tübingen, Germany; 4 Department of Dermatology, Venereology and Allergology, Kantonsspital St Gallen, Sankt Gallen, Switzerland; 5 Department of Computer Science, Eberhard Karls University of Tübingen, Tübingen, Germany; 6 Skin Cancer Center at the University Cancer Centre and National Center for Tumor Diseases Dresden; Department of Dermatology, Faculty of Medicine and University Hospital Carl Gustav Carus, Technische Universität Dresden, Dresden, Germany; 7 Department of Dermatology, University of Lübeck, Lübeck, Germany; 8 Charité – Universitätsmedizin Berlin, corporate member of Freie Universität Berlin and Humboldt-Universität zu Berlin, Department of Dermatology, Venereology and Allergology, Berlin, Germany; 9 Department of Immunology, Interfaculty Institute for Cell Biology, Eberhard Karls University Tübingen, Tübingen, Germany; 10 Health Sciences North Research Institute, Sudbury, Ontario, Canada

**Keywords:** melanoma, biomarkers, tumor, immunotherapy, myeloid-derived suppressor cells, T-lymphocytes

## Abstract

**Background:**

The need for reliable clinical biomarkers to predict which patients with melanoma will benefit from immune checkpoint blockade (ICB) remains unmet. Several different parameters have been considered in the past, including routine differential blood counts, T cell subset distribution patterns and quantification of peripheral myeloid-derived suppressor cells (MDSC), but none has yet achieved sufficient accuracy for clinical utility.

**Methods:**

Here, we investigated potential cellular biomarkers from clinical routine blood counts as well as several myeloid and T cell subsets, using flow cytometry, in two independent cohorts of a total of 141 patients with stage IV M1c melanoma before and during ICB.

**Results:**

Elevated baseline frequencies of monocytic MDSCs (M-MDSC) in the blood were confirmed to predict shorter overall survival (OS) (HR 2.086, p=0.030) and progression-free survival (HR 2.425, p=0.001) in the whole patient cohort. However, we identified a subgroup of patients with highly elevated baseline M-MDSC frequencies that fell below a defined cut-off during therapy and found that these patients had a longer OS that was similar to that of patients with low baseline M-MDSC frequencies. Importantly, patients with high M-MDSC frequencies exhibited a skewed baseline distribution of certain other immune cells but these did not influence patient survival, illustrating the paramount utility of MDSC assessment.

**Conclusion:**

We confirmed that in general, highly elevated frequencies of peripheral M-MDSC are associated with poorer outcomes of ICB in metastatic melanoma. However, one reason for an imperfect correlation between high baseline MDSCs and outcome for individual patients may be the subgroup of patients identified here, with rapidly decreasing M-MDSCs on therapy, in whom the negative effect of high M-MDSC frequencies was lost. These findings might contribute to developing more reliable predictors of late-stage melanoma response to ICB at the individual patient level. A multifactorial model seeking such markers yielded only MDSC behavior and serum lactate dehydrogenase as predictors of treatment outcome.

**WHAT IS KNOWN ON THIS TOPIC**

- So far, serum lactate dehydrogenase (LDH) levels are the only established, clinically used biomarkers for predicting the prognosis of metastatic melanoma.
- However, high peripheral blood baseline monocytic myeloid-derived suppressor cell (M-MDSC) frequencies prior to immunotherapy are consistently statistically significantly associated with worse clinical outcome in many studies.

**WHAT THIS STUDY ADDS**

- We confirm that elevated M-MDSC frequencies are associated with shorter survival on average, and describe a subset of patients whose high baseline M-MDSC frequencies decrease early during immunotherapy.
- These patients have an overall survival (OS) similar to those initially with low MDSC frequencies.
- Furthermore, we establish a multifactor model associated with OS which includes serum LDH levels and MDSC behavior.

**HOW THIS STUDY MIGHT AFFECT RESEARCH, PRACTICE OR POLICY:**

- This study shows that monitoring of peripheral blood M-MDSC frequency during therapy provides a method for the early identification of patients responding to immunotherapy versus those who might benefit more from other therapies.

## Background

Over the last decade, there have been great improvements in the survival of patients with advanced melanoma due to the use of immune checkpoint inhibitors targeting the cytotoxic T-lymphocyte antigen-4 (CTLA-4) and programmed cell death protein 1 (PD-1) on the surface of activated T cells.^1 2^ However, although many patients experience at least temporary clinical benefits, a subset does not respond or develops resistance to such immune-checkpoint blockade (ICB).^3^ Thus, the identification of specific biomarkers predicting response, ideally derived non-invasively from peripheral blood, remains an important clinical need. The serum lactate dehydrogenase (LDH) level is the only established biomarker in melanoma so far.^4 5^ However, other parameters like routine blood counts,^6–8^ circulating tumor DNA,^9^ frequency of activated monocytes,^10^ defined T cell subsets or myeloid-derived suppressor cells (MDSCs) have been suggested as promising candidates.^6 11–15^ While molecular mechanisms of therapy response or resistance to ICB are not yet fully understood, the inhibition or acceleration of the activity of certain T cell subsets in the periphery and tumor microenvironment seems to be of great importance for clinical outcome.^16–18^ Suppression of T cells by MDSCs contributes to profound immune dysfunction^19 20^ and cancer immune evasion strategies,^21^ mediated mostly, but not exclusively by monocytic MDSCs (M-MDSC) which are the largest subset. High peripheral blood frequencies of M-MDSCs are more prevalent in patients with melanoma than in healthy donors,^22^ and correlate with clinical cancer stages,^23^ and are associated with tumor development and progression.^24^

Here, we screened defined myeloid and T cell subsets in the blood of patients with late-stage melanoma before initiating and early during ICB to investigate potential associations with clinical outcome. We confirm the well-known general finding of ourselves and others that highly elevated frequencies of peripheral M-MDSCs associate significantly but not very closely with poorer outcomes of ICB in metastatic melanoma at the population level.^6 13 23–25^ However, we find that in a subgroup of patients with rapidly decreasing M-MDSCs early during therapy, the poor prognosis associated with high baseline (BL) M-MDSC frequencies is lost, increasing the possibility of using levels of these cells as dynamic biomarkers of clinical outcome at the individual patient level.

## Methods

### Patients

Only patients with stage IV melanoma classified by the site of metastasis and their LDH level as M1c according to American Joint Committee on Cancer (AJCC) 2009^5^ were included in this study to investigate peripheral blood immune correlates with clinical outcome at a very late stage of disease. For the discovery cohort, we used cryopreserved peripheral blood mononuclear cells (PBMCs) from venous EDTA blood drawn before start of immunotherapy (baseline, BL) and a median of 44 days thereafter (follow-up (FU)) from 92 patients with melanoma treated between May 2015 and March 2017 at clinical centers in Dresden, Lübeck and Tübingen. For the validation cohort, samples from 49 patients were obtained from our biobank and derived also from the centers in Dresden and Tübingen from April 2017 to October 2019 following our established protocols. Clinical routine hemograms determined before or on the day of sampling (IQR 0–3 days) of the respective time point were used for analyses in this study. The cohorts investigated here overlap partially with cohorts from earlier published studies.^26 27^ Detailed patient characteristics are given in table 1.

**Table 1:** Patients’ characteristics

Factor	Category	Discovery		Validation	
		N	%	N	%
Age	Median	67		64	
	IQR	21.5		22	
Sex	Female	34	37.0	18	36.7
	Male	58	63.0	31	63.3
Center	Tübingen	67	72.8	42	85.7
	Dresden	17	18.5	7	14.3
	Lübeck	8	8.7	0	0.0
Therapy	**Anti-PD-1**	**51**	**55.4**	**14**	**28.6**
	2 mg/kg once every 3 weeks Pembro	42	45.7	8	16.3
	3 mg/kg once every 3 weeks Pembro	3	3.3	0	0.0
	4 mg/kg once every 6 weeks Pembro	1	1.1	0	0.0
	400 mg once every 6 weeks Pembro	0	0.0	1	2,0
	3 mg/kg once every 2 weeks Nivo	5	5.4	0	0.0
	240 mg once every 2 weeks Nivo	0	0.0	1	2.0
	480 mg once every 4 weeks Nivo	0	0.0	4	8.2
	**Anti-PD-1+anti-CTLA-4**	**41**	**44.6**	**35**	**71.4**
	1 mg/kg Ipi+3 mg/kg Nivo once every 3 weeks	4	4.3	3	6.1
	3 mg/kg Ipi+1 mg/kg Nivo once every 3 weeks	37	40.2	32	65.3
Previous systemic therapies	Immunotherapy	20	21.7	15	30.6
	Targeted therapy	22	23.9	10	20.4
	Chemotherapy	4	4.3	4	8.2
	None	56	60.9	26	53.1
LDH serum level BL	Normal	42	45.7	20	40.8
	Elevated	50	54.3	27	55.1
	Unknown	0	0.0	2	4.1

BL, baseline; CTLA-4, cytotoxic T-lymphocyte-associated protein 4; Ipi, ipilimumab; LDH, lactate dehydrogenase; n.a., not available; Nivo, nivolumab; PD-1, programmed cell death protein 1; Pembro, pembrolizumab.

### Flow cytometry

Patient samples were thawed in batches and stained with monoclonal antibody panels to characterize myeloid and T cells as described previously.^27^ In brief, in the myeloid cell panel, dead cells were excluded using ethidium monoazide bromide (EMA, Biotinum) with simultaneous blockade of free Fcγ-receptors (Gamunex, Grifols) and stained for extracellular cell surface markers (online supplemental table 1). M-MDSC were defined as viable lineage-negative (CD3-CD19-CD56−) cells expressing CD11b, CD33 and CD14 but little or no HLA-DR. Monocytes were defined as viable lineage-negative CD11b+CD33+HLA-DR+ cells and are further subcategorized by their different CD14 and CD16 expression. Classical monocytes were defined as CD14+CD16−, intermediate monocytes as CD14+CD16+ and non-classical monocytes as CD14dimCD16+ cells (online supplemental figure 1). To investigate T cells and their checkpoint expression, two aliquots of the same PBMC samples that were used for the myeloid cell panel were concurrently stained. After staining of dead cells (EMA, Biotinum) and blockade of free Fcγ-receptors (Gamunex, Grifols), samples were incubated with antibodies against cell surface markers. Both aliquots were stained with antibodies against CD3, CD4, CD8, CD25 and CD127. For investigation of checkpoint expression, cells were either stained with antibodies against PD-1, lymphocyte activation gene 3 (LAG-3) and T cell immunoglobulin and mucin-domain containing-3 (TIM-3) or with their respective isotype controls. To identify regulatory T cells (Tregs), the samples were fixed and permeabilized using the FoxP3 Transcription Factor staining buffer set (Thermo Fisher Scientific) and then incubated with FoxP3 antibodies (online supplemental table 1). T cells were defined as CD3+ viable lymphocytes and further subdivided with antibodies against CD4 and CD8 to detect the major T cell subsets in addition to Tregs identified as CD25+CD127low/-FoxP3+CD4+ T cells. Checkpoint expression was defined as the fraction of positive cells within the parental T cell subset.

10.1136/jitc-2023-006802.supp1 Supplementary data

The samples were acquired immediately after staining on an LSR II cytometer (BD) and data analyzed using FlowJo (V.10.7.1, BD) using established gating strategies (online supplemental figures 1 and 2).

### Statistical analyses

Overall survival (OS) was defined as the time from the start of therapy until death or last patient contact. Other causes of death were regarded as censored events. Progression-free survival (PFS) was defined as the time between the start of therapy and disease progression or last FU. Response was evaluated using RECIST V.1.1 criteria.^28^ OS and PFS probabilities were calculated and analyzed using the Kaplan-Meier approach and log-rank testing (Prism, V.5.0e; GraphPad Software). Neutrophil-to-lymphocyte ratios (NLR) were calculated by the absolute numbers of neutrophils relative to lymphocytes in fresh blood counts, and the LDH ratio was calculated by the LDH serum level relative to the upper limit of normal (ULN).

To investigate potential associations between cellular parameters and OS before the start of therapy, we used a minimized cut-off screening approach, similar to that published earlier.^6 13 29^ In brief, the cellular features at BL of the first cohort were divided into three equal subsets (<33 percentile, 33–66 percentile and >66 percentile) to identify two cut-off points for the dichotomization of the BL samples to consider extreme values in addition to the median values. Features and cut-off points that correlated with patients’ OS in the discovery cohort were then validated or rejected in an analysis of the second cohort. The resulting p values were corrected for multiple testing using the Bonferroni method, and throughout this study p<0.05 was considered statistically significant.

BL and FU samples were compared using Wilcoxon matched-pairs signed rank testing, and groups by the Mann-Whitney U test (Prism V.5.0e; GraphPad Software). To visualize cell populations, violin plots were created using the packages ggplot2 and introdataviz in R studio (R V.4.2.0 in R studio V.1.1.463). For the statistical evaluation of more than two groups, one-way analysis of variance (Kruskal-Wallis test) with Dunn’s post hoc testing was performed. Correlations of features were evaluated using the Spearman’s R test.

The Cox proportional hazards regression model and the resulting forest plots were calculated using the survminer and the survival package^30^ using R studio (R V.4.2.0 in R studio V.1.1.463). For multivariate Cox proportional hazards regression modeling, patients with missing data in the variables were excluded from the multivariate analysis.

## Results

### Patients

In total, 141 patients with stage IV melanoma were recruited in daily clinical practice at the centers in Tübingen, Dresden and Lübeck, to investigate peripheral blood-derived cellular biomarker candidates informative for clinical outcome. Ninety-two patients were included in the discovery cohort, and 49 in the validation cohort (median age: 67 (IQR 55–76) years and 64 (IQR 53–75) years, respectively). In both cohorts, there were more male than female patients (discovery 63% vs 37% and validation 63.3% vs 36.7%, respectively). Patients received either anti-PD-1 antibodies alone or in combination with anti-CTLA-4 antibodies. Because of the real-world study setting, although the number of patients in the discovery cohort receiving either therapy was similar (55.4% vs 44.6%, respectively), the validation cohort collected at a later time point included significantly more patients treated with the antibody combination (71.4% vs 28.5%, p=0.003, Fisher’s exact test). Nonetheless, there was no significant difference in the OS of patients between the two treatments, age or sex in both cohorts (online supplemental figure 3A,B). In the discovery and validation cohorts, 60.9% and 53.1% of patients had not received any previous systemic therapy. Detailed patient characteristics are provided in table 1 and the treatment and sampling scheme is summarized in online supplemental figure 4. The 2-year OS of the discovery cohort was 37.5% and of the validation cohort 58.9%, and 2-year PFS was 14.1% and 22.8%, respectively.

### Highly elevated baseline M-MDSC frequencies correlate with poor survival

**Figure 1:**
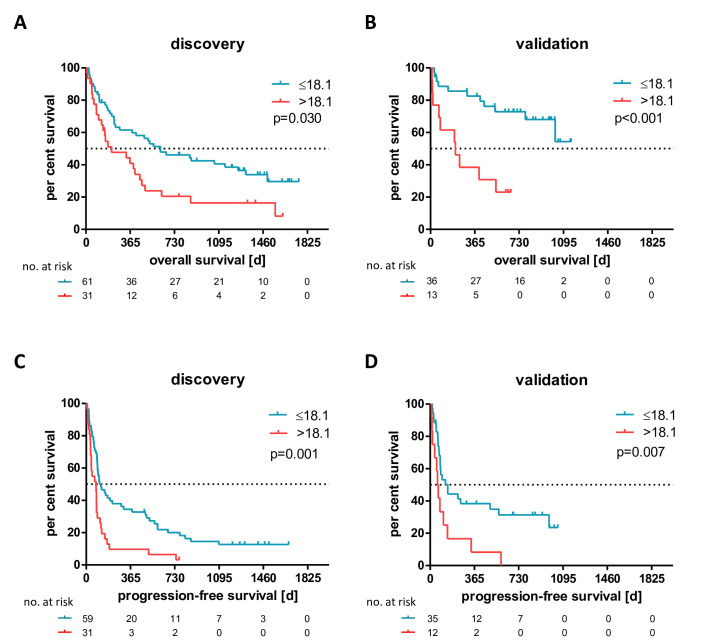
High monocytic myeloid-derived suppressor cell frequencies before start of immune checkpoint blockade correlate significantly with shorter overall and progression-free survival in the discovery (A: p=0.030 and C: p=0.001) and validation cohort (B: p<0.001 and D: p=0.007).

Thirty-three cellular features were determined from routine blood counts and in-depth immunophenotyping investigations, including frequencies of cells with M-MDSC, monocyte, CD4+, CD8+ and Treg cell phenotypes in blood samples drawn before the start of therapy (BL) from patients of the discovery cohort. To identify potential correlations of these with patients’ OS, we stratified the discovery cohort according to the median value and two additional cutoffs (<33 and >66 percentile) per cellular feature, similar to approaches that have been published before.^6 13 29^ Of all investigated cellular variables, only the upper cut-off values of absolute basophil counts (0.07×1000 cells/µL) and M-MDSC frequencies of total mononuclear leucocytes (>18.1%) were selected as predictive candidate features for patients’ OS. Only the latter could be validated in an independent analysis of the second cohort (online supplemental tables 2–4). Thus, patients with a BL M-MDSC frequency >18.1% (‘M-MDSC-high’) had on average a significantly shorter OS than those with a frequency ≤18.1% (‘M-MDSC-low’) in both the discovery and validation cohorts (HR 2.086, p=0.030; HR 7.652, p<0.001, figure 1A,B, respectively). These findings were independent of the PD-1 therapy received (online supplemental figure 5). M-MDSC-high patients also experienced significantly shorter PFS in the discovery and in the validation cohort (HR 2.425, p=0.001 and HR 3.344, p=0.007, respectively, figure 1C,D). Potential confounding factors such as type of therapy (monotherapy vs combination therapy) (online supplemental figure 6), previous therapies (online supplemental figure 7), age, LDH, S100 serum levels (online supplemental figure 8) or centers (online supplemental figure 9) did not associate significantly with the determined M-MDSC frequencies. Moreover, the predictive biomarker characteristics of the M-MDSC frequencies before the start of therapy for the clinical outcome under therapy correlated independently of the only biomarker established in the clinic, the LDH levels, with patients’ OS in a multivariate Cox regression analysis (online supplemental figure 10).

### M-MDSC-high patients display a skewed peripheral immune cell signature

**Figure 2:**
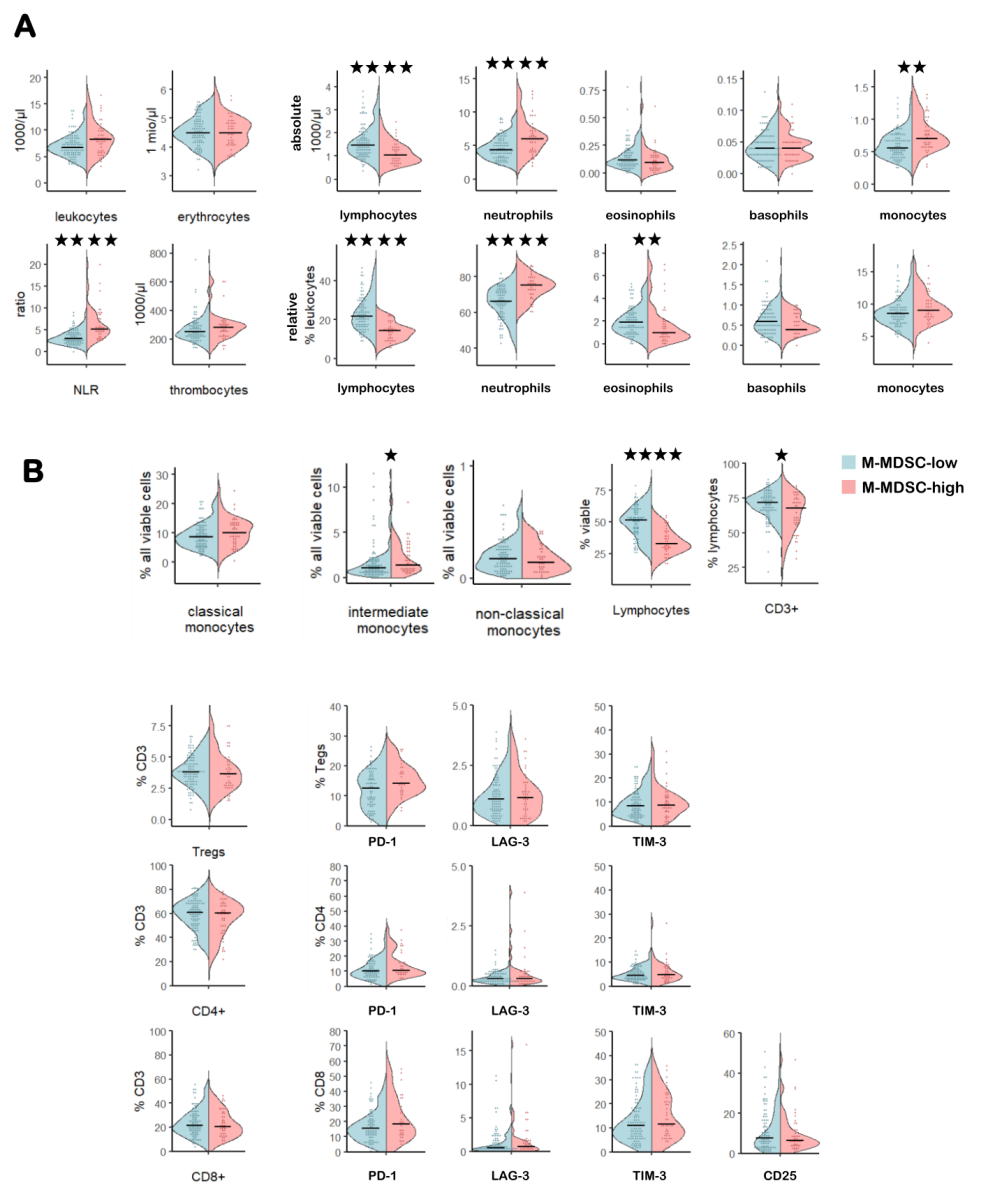
Comparison of the peripheral immune signature in M-MDSC-high versus M-MDSC-low patients. Violin plots depicting blood counts from the hemogram (A) and flow cytometry-derived frequencies of myeloid cells, T cells and their checkpoint receptor expression (B). *P<0.05, **p<0.01, ***p<0.005, ****p<0.0001. LAG-3, lymphocyte activation gene 3; M-MDSC, monocytic myeloid-derived suppressor cell; NLR, neutrophil-to-lymphocyte ratio; PD-1, programmed cell death protein 1; TIM-3, T cell immunoglobulin and mucin-domain containing-3; Treg, regulatory T cell.

Combined analyses of both cohorts revealed that the peripheral immune cell signature of M-MDSC-high patients, although not informative for survival in univariate analyses, had a skewed composition at BL. Thus, M-MDSC-high patients had significantly lower lymphocytes (blood counts and flow cytometry investigations), CD3+ T cells and eosinophils (relative and absolute number), but higher absolute monocytes (presumably caused by the highly elevated M-MDSC frequencies), higher relative and absolute neutrophil counts, intermediate monocytes, PD-1+Tregs and a higher NLR compared with M-MDSC-low patients (figure 2 and online supplemental table 5).

**Figure 3:**
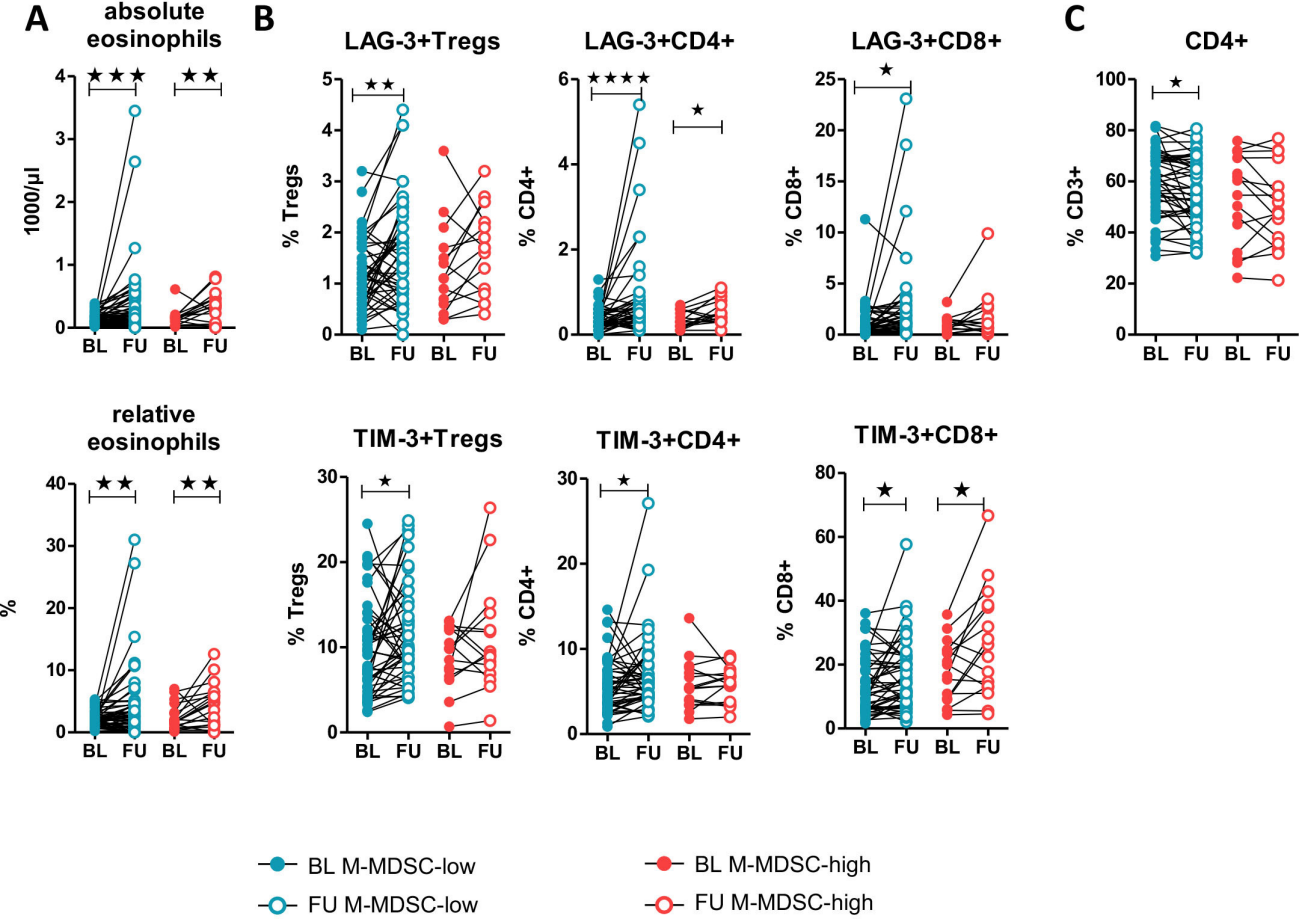
Comparison of significant changes in the abundance of immune cell subsets under immune checkpoint blockade in M-MDSC-high versus M-MDSC-low patients: eosinophils (A), checkpoint receptor expression on T cells (B) and total CD4+ T cell frequencies (C). *P<0.05, **p<0.01, ***p<0.005, ****p<0.0001. BL, baseline; FU, follow-up; LAG-3, lymphocyte activation gene 3; M-MDSC, monocytic myeloid-derived suppressor cell; TIM-3, T cell immunoglobulin and mucin-domain containing-3.

**Figure 4:**
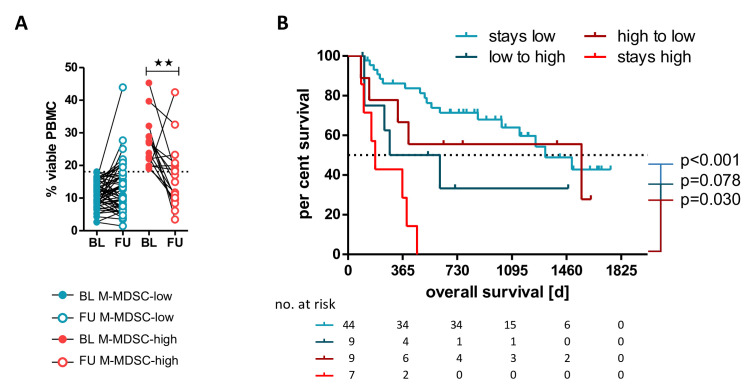
Comparison of changes in the M-MDSC frequencies in M-MDSC-low versus M-MDSC-high patients under immune checkpoint blockade. Patients in the M-MDSC-high group had a significant decrease in their M-MDSC frequencies (A, p=0.010). Those patients whose M-MDSC frequency stayed high had a significantly shorter overall survival compared with those with a decrease or an M-MDSC frequency that stayed low (B). **P<0.01. BL, baseline; FU, follow-up; M-MDSC, monocytic myeloid-derived suppressor cell; PBMC, peripheral blood mononuclear cell.

Significant signature composition changes early under therapy were seen in M-MDSC-high and M-MDSC-low patients as follows: increased absolute and relative eosinophils (figure 3A), and LAG-3+CD4+, TIM-3+CD4+ and LAG-3+CD8+ T cells (figure 3B). In contrast, increased CD4+ T cells (figure 3C), LAG-3+ and TIM-3+Tregs, TIM-3+CD4+ and LAG-3+CD8+ T cells (figure 3B) and absolute leukocyte and relative lymphocyte counts (online supplemental figure 11A) were only present in the M-MDSC-low patients. Interestingly, there were no significant changes of the frequency of total lymphocytes, CD3+ and CD8+ T cells, Tregs and monocyte subsets in either group (online supplemental figure 11CB+) but some of the patients in the M-MDSC-high group had a significant decrease in their M-MDSC frequency under ICB (p=0.010, figure 4A). Despite these differences in the frequencies of the investigated immune cells, except for the MDSCs, there were no associations of these features with OS, as described in the previous section.

### A fraction of M-MDSC-high patients does benefit from anti-PD-1 ICB

Based on the identified changes of M-MDSC frequencies early under therapy, we developed a model to investigate the potential impact of these changes on the identified biomarker cut-off value of 18.1% in a combined analysis of both cohorts. Because no differences were found between patients receiving PD-1 monotherapy or a combination with anti-CTLA-4 antibodies, patients who received either PD-1 antibodies alone or in combination with CTLA-4 antibodies were examined here, as before. As expected, patients with M-MDSC frequencies ≤18.1% before and under therapy had in general a superior OS compared with those with frequencies >18.1% at both time points (HR 0.004, p<0.001, figure 4B). However, those M-MDSC-high patients whose M-MDSC frequency declined to below 18.1% under ICB had a prolonged OS relative to those whose M-MDSC frequency remained high early during treatment (HR 4.133, p=0.030, figure 4B). No significant difference in OS was found for patients with M-MDSC frequencies exceeding the cut-off of 18.1% at FU, compared with those with values >18.1% at both time points (HR 0.331, p=0.078). The 2-year survival rate for patients whose M-MDSC frequency did not decline to under 18.1% was 0%, and for patients with increasing M-MDSC frequencies (low to high) it was 33.3%. In contrast, for patients with M-MDSC frequencies falling to below the cut-off (high to low), it was 55.6% and for patients with a BL M-MDSC frequency below the cut-off and for low-MDSC patients remaining low it was 71.4%.

### Determination of a risk factor model

**Figure 5:**
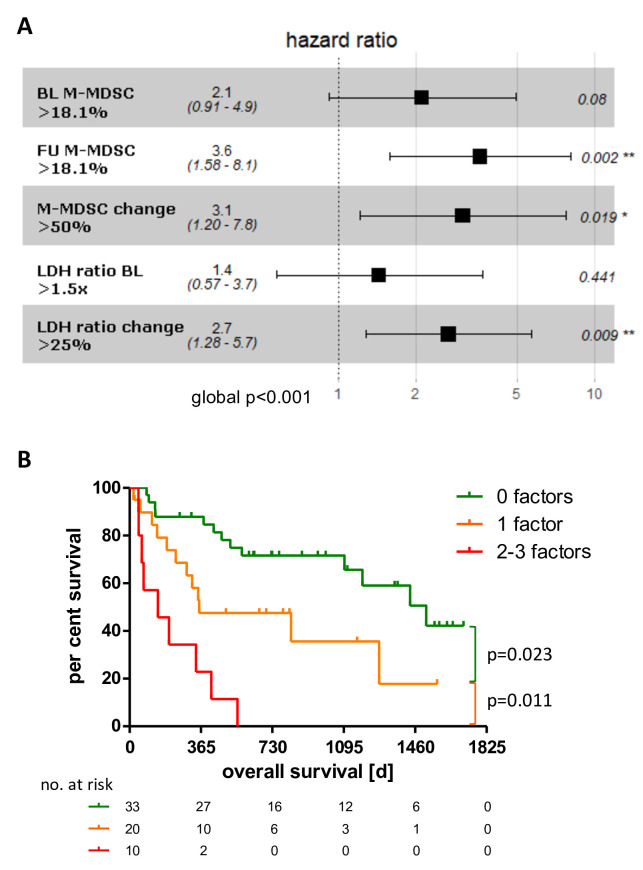
Multivariate modeling of biomarker candidates that correlate significantly with overall survival in univariate analysis. Results of multivariate Cox regression (A). The significantly independent features of the multivariate Cox regression have been used to compute a combinatorial predictive biomarker model (B). *P<0.05, **p<0.01. BL, baseline; FU, follow up; LDH, lactate dehydrogenase; M-MDSC, monocytic myeloid-derived suppressor cell.

To evaluate the identified biomarker properties of highly elevated M-MDSC, we tested their dependency on the only established biomarker, the LDH serum level, as these two parameters were the only ones to show significant associations with OS. Univariate analysis of the combined cohorts revealed a negative correlation of highly elevated M-MDSC frequencies with patients’ OS at BL (HR 2.86, p<0.001, online supplemental figure 12A). A 1.5-fold elevated BL LDH ratio, which was described previously as clinically meaningful,^31^ correlated with shorter survival (HR 1.98, p=0.014, online supplemental figure 12B). In addition, a general increase of >25% of the LDH ratio determined at BL was indicative of patients with shorter OS (HR 4.00, p=0.004, online supplemental figure 12C). Patients with M-MDSC frequencies >18.1% at FU, or those with a marked increase of >50% (>1.5-fold) also had significantly shorter OS (HR 6.97, p<0.001 and HR 3.08, p=0.026, respectively, online supplemental figure 5D,E). To investigate dependencies of those features that correlated in univariate analyses significantly with patients’ OS, we performed multivariate Cox regression analysis including in addition to the BL features serum LDH ratio (>1.5×ULN) and M-MDSC frequencies (>18.1%). We identified highly elevated M-MDSC FU frequencies (HR 3.581, p=0.002), a general increase of M-MDSCs (HR 3.060, p=0.019) and LDH levels (HR 1.441, p=0.009) as independent significant markers for poor OS with a global p<0.001 (figure 5A). A combinatorial model of these three independent markers was constructed, where patients were stratified according to the sum of risk factors (figure 5B). The more risk factors a patient accumulated, the shorter was the OS. The 2-year survival rate for patients with zero risk factors was 72%, for patients with at least one risk factor was 48% and for those with two to three risk factors was 0%.

## Discussion

Here, employing a validated multicentric study, we sought more informative biomarker candidates derived from routine blood counts and peripheral T cell and myeloid cell subset phenotyping in patients with advanced stage IV melanoma. We found that of all parameters analyzed, only extremely elevated frequencies of >18.1% of circulating CD33+CD11b+CD14+HLA-DRlow/− M-MDSC before and under anti-PD-1±anti-CTLA-4 ICB were significantly associated with shorter OS. Elevated M-MDSC frequencies are a well-known cellular biomarker candidate that has been repeatedly reported to correlate negatively with OS in patients treated with anti-PD-1 monotherapy^32–34^ or anti-CTLA-4 monotherapy.^6 35^ However, somewhat in contrast to previous studies with M-MDSC cut-off values of ~10%–13%,^33 34^ in the present study we identified this negative association only in patients with very highly elevated M-MDSC frequencies (>18.1%). Similar to our findings, a recent study using a machine learning-based myeloid index score in patients with melanoma with mixed treatments found that only the very high-frequency percentiles of 85% of M-MDSC were informative for higher risk of progression and shorter OS.^36^ These findings may be due to the evolving clinical routine (first-line) treatment using ICB, implying that patients with intermediate MDSC frequencies may now also be benefiting from this therapy. Although quantification of M-MDSCs is non-standardized and very likely dependent on technical factors in different centers, the clinical relevance of the cutoffs in this and other current studies^36^ compared with previous studies^6 33–35^ is consistent.

Despite the fact that many immune cell subsets examined do not correlate with patients’ OS in univariate analyses, examination of the composition of the peripheral immune signature of M-MDSC-low and M-MDSC-high patients revealed differences between them. We found that the latter had a skewed immune cell signature including cellular features already associated with impaired cancer immunosurveillance in some studies, such as fewer lymphocytes, CD3+ T cells and eosinophils and a higher NLR. The latter, for example, is used as a measure of an impaired immune system and has been associated with worse clinical outcomes across several cancer types, including melanoma.^37 38^ Several effects of ICB observed here are consistent with published studies, such as an increase in absolute and relative eosinophils, which has already been reported for patients with melanoma treated with anti-CTLA-4 antibodies.^39^ Even though we did not find correlations of T cells with survival, we observed increases in frequencies of LAG-3-positive and TIM-3-positive cells within CD4+ and CD8+ T cell populations in M-MDSC-low patients. These increases might be the result of an ICB-induced activation of alternative regulatory pathways. They could thus be a sign of T cell response to therapy and presumably not of an increase of T cell inactivity.^40 41^

Along those lines, M-MDSC-high patients with poor clinical outcome revealed only an increase of checkpoint receptor-positive populations in a few T cell subsets, supporting the hypothesis of an immune-compromised status of these patients. However, the majority of these patients had a significant decrease in M-MDSC frequencies under ICB, suggesting a therapy-associated modulatory effect. Of particular interest was the finding that those M-MDSC-high patients that experienced an early decrease of M-MDSC frequencies down to below the cut-off of 18.1% had a superior clinical benefit from ICB, similar to those with an initially low M-MDSC frequency. To the best of our knowledge, published studies have so far only revealed negative associations of MDSC frequencies under CTLA-4^6 42^ and PD-1^34^ therapies with patients’ OS, but there have been no reports on changes of MDSC frequencies under therapy of patients who were initially assigned to a poor prognosis group. These patients are characterized by a significant reduction of M-MDSC frequencies early under therapy. However, not only the categorical dichotomization of M-MDSC frequencies according to the identified 18.1% cut-off but also a strong increase of their frequencies early under ICB (independent of the BL value) correlated negatively with patients’ OS. This was independent of the established biomarker LDH (risk factor model) and thus complements earlier published findings. For example, similar associations of clinical benefit with a decrease of MDSCs 3 weeks^42^ and 6 weeks^43^ after starting anti-CTLA-4 therapy were previously described using univariate analysis in patients with melanoma. Also, in non-small cell lung cancer and urothelial carcinoma under anti-PD-1 ICB, decreased M-MDSC frequencies were associated with better clinical responses.^44 45^ The involvement of PD-1 blockade in differentiation and development of M-MDSCs in humans is still unknown. However, mice deficient for PD-1 have fewer M-MDSCs suggesting that PD-1 contributes presumably to the differentiation of M-MDSCs.^46 47^ If PD-1 contributes to MDSC differentiation, then PD-1 blockade might be responsible for the decrease of M-MDSC observed in our study. Future studies of matched samples of peripheral blood and tumor tissue will be required to (i) determine possible kinetics under therapy and to (ii) provide further insights into the role of MDSCs in ICB, because available data suggest that the circulating levels of MDSCs mirror those within the tumor microenvironment.^48^

There are limitations to this study that need to be considered, particularly the combined analysis of patients receiving anti-PD-1 antibodies alone or in combination with anti-CTLA-4. There is a body of data describing different mechanisms and consequently different immune cell phenotypes as being relevant for one or the other treatment strategy.^14 49 50^ Unfortunately, our cohort size did not allow a comparative analysis, but the M-MDSC data and the associated changes in the immune cell signature under ICB are so clear that it is unlikely that the results are relevant only to one treatment strategy.

In conclusion, our data suggest that contrary to the general consensus, possessing pre-existing highly elevated frequencies of peripheral M-MDSC before ICB does not accurately identify all patients who fail to benefit from treatment. Rather, the dynamic change under therapy serves as a better predictor of clinical benefit in patients with metastatic melanoma. Understanding the mechanisms responsible for these changes in some but not other patients with high BL MDSC levels should facilitate rational interventions to increase the proportion of clinically responsive patients.

## Data Availability

Data are available on reasonable request.
