# Correction: Early decrease of blood myeloid-derived suppressor cells during checkpoint inhibition is a favorable biomarker in metastatic melanoma

**DOI:** 10.1136/jitc-2023-006802corr1

**Published:** 2023-09-19

Gaißler A, Bochem J, Spreuer J, *et al*. Early decrease of blood myeloid-derived suppressor cells during checkpoint inhibition is a favorable biomarker in metastatic melanoma. *Journal for ImmunoTherapy of Cancer* 2023;**11:**e006802. doi: 10.1136/jitc-2023-006802

The licence was updated to CC-BY in September 2023. The funding statement has been updated to include the following "we acknowledge support from the Open Access Publishing Fund of the University of Tübingen".

