# Supplementary material for: Early decrease of blood myeloid-derived suppressor cells during checkpoint inhibition is a favorable biomarker in metastatic melanoma
Source: J Immunother Cancer. 2023 Jun 7;11(6):e006802. doi: 10.1136/jitc-2023-006802 (PMC10254874; doi:10.1136/jitc-2023-006802)
Supplement: Supplementary data [file jitc-2023-006802supp001.pdf]

Early decrease of blood myeloid-derived suppressor cells during checkpoint inhibition is a favourable biomarker in metastatic melanoma

Andrea Gaißler*[1,2], Jonas Bochem*[1,2], Janine Spreuer[1,2], Shannon Ottmann[1], Alexander Martens[1], Teresa Amaral[1,3], Nikolaus Benjamin Wagner[1,4], Manfred Claassen[2,5], Friedegund Meier[6], Patrick Terheyden[7], Claus Garbe[1], Thomas Eigentler[8], Benjamin Weide[1], Graham Pawelec[9,10], Kilian Wistuba-Hamprecht[1,2,9]

*Shared first authorship

Supplementary Information

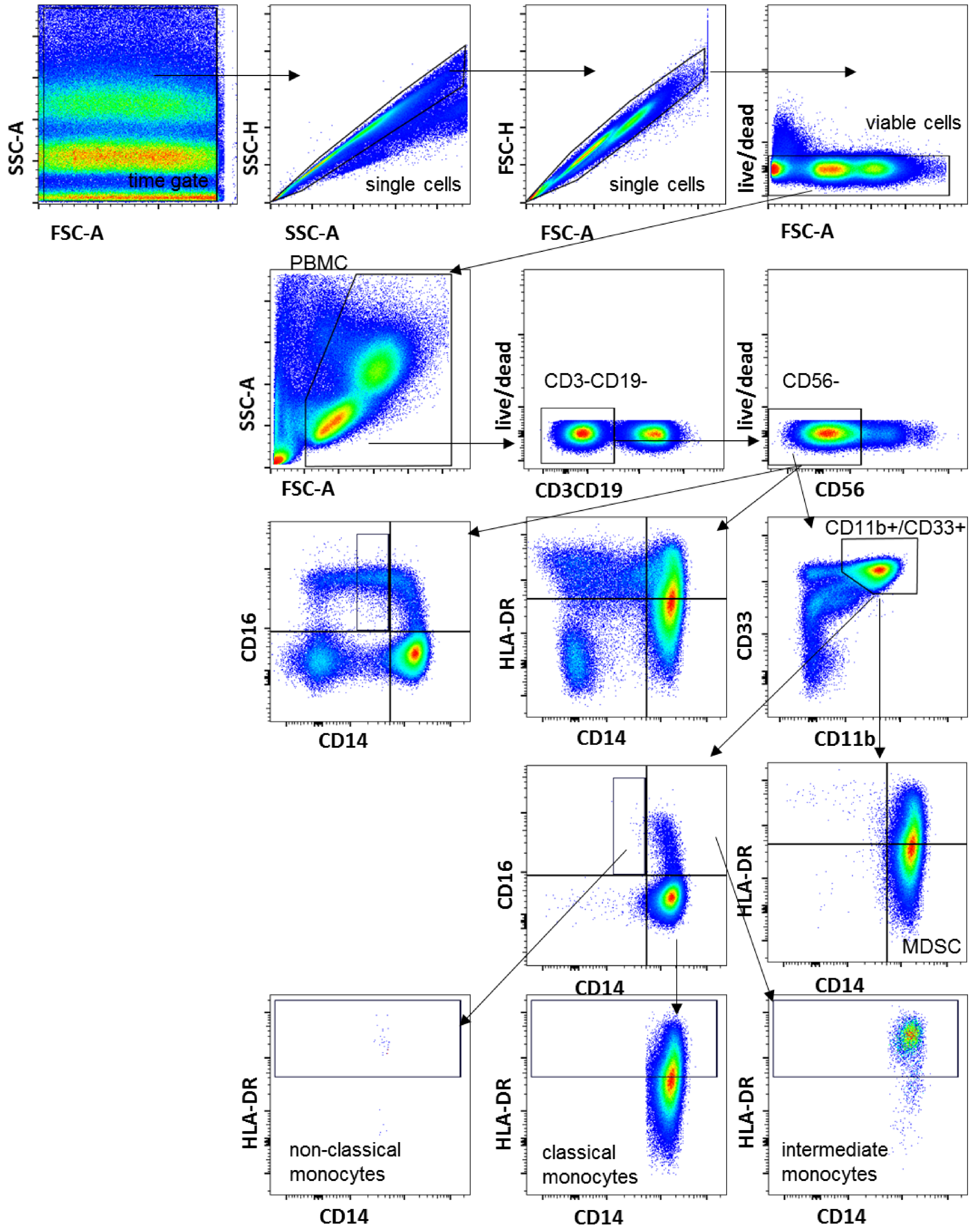

Supplementary Figure 1: Gating strategy of the myeloid cell panel. Adjustment of the gates for MDSCs and CD14+CD16-, CD14+CD16+ and CD14dimCD16+ cells were performed on the CD56- population and then copied to the CD11b+CD33+ population. M-MDSC frequencies were calculated to all assessed leukocytes within the PBMC gate.

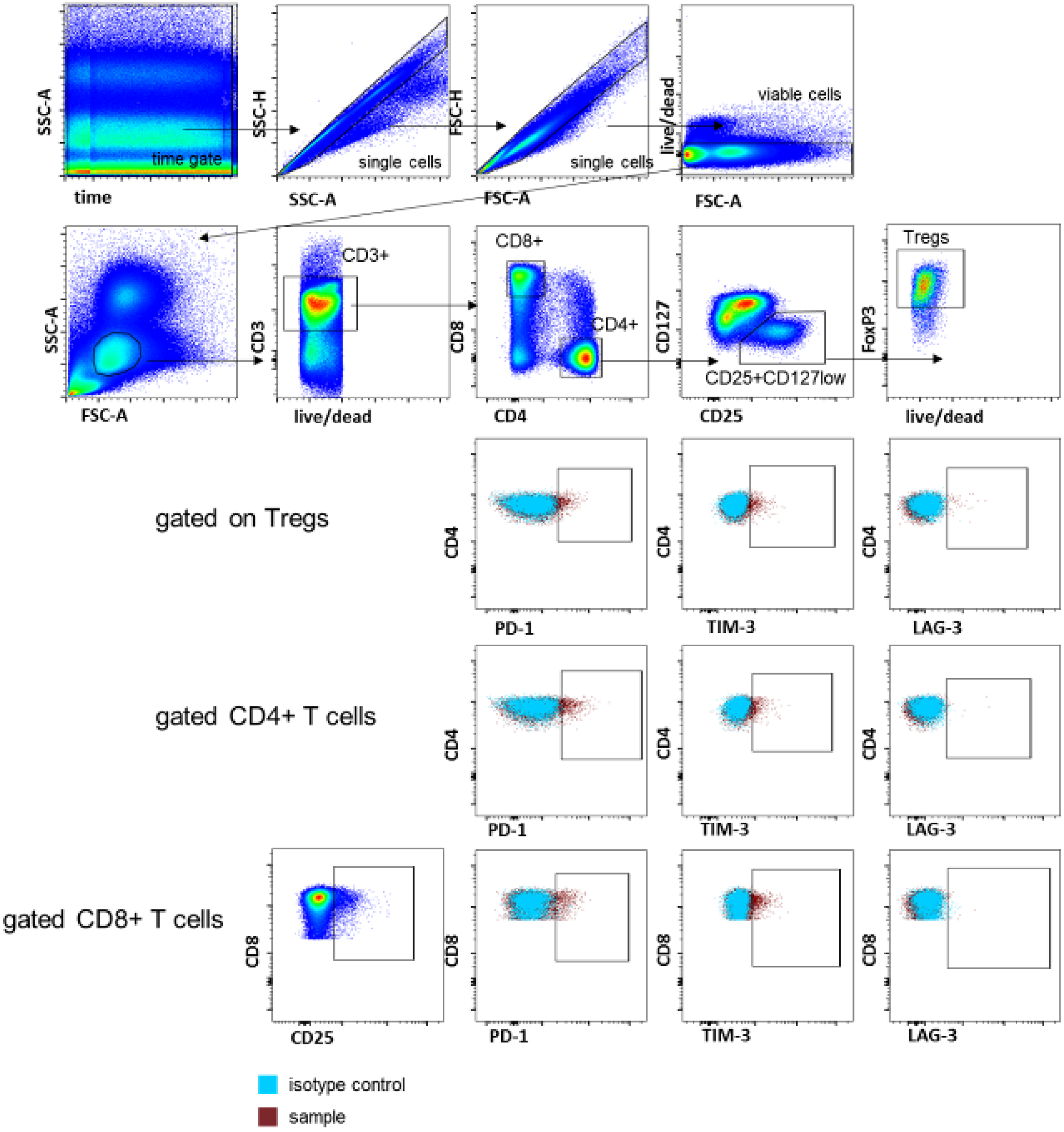

Supplementary Figure 2: Gating strategy of the T cell-panel. Gates for the checkpoint molecules were set on the isotype control (blue) and then copied to the sample (red).

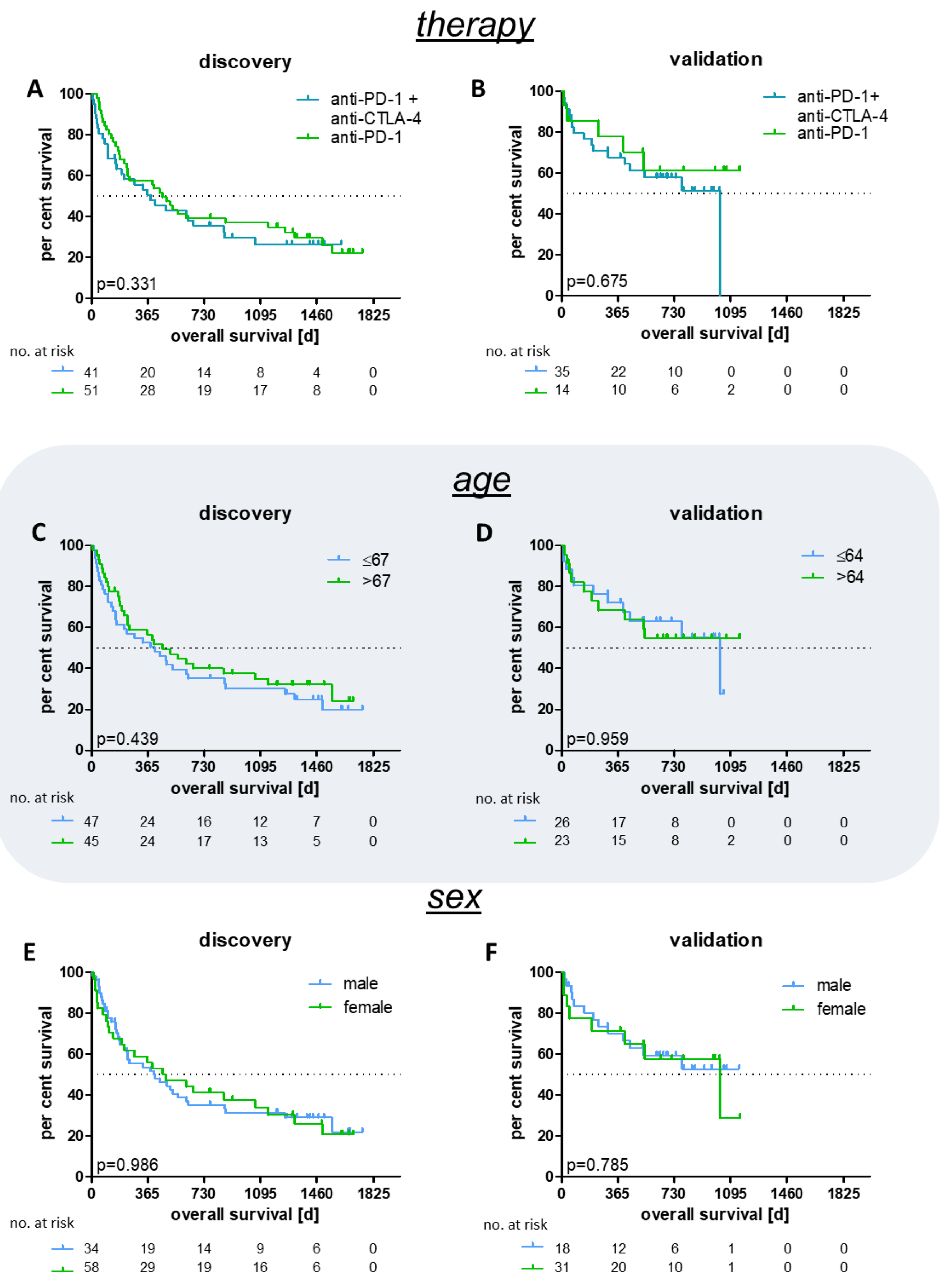

Supplementary Figure 3: Investigation of potential correlations of therapy (A: p=0.331 and B. p=0.650), age (C: p=0.439 and D: p=0.959) and sex (E: p=0.986 and F: p=0.785) with overall survival in the discovery and validation cohort using log-rank testing.

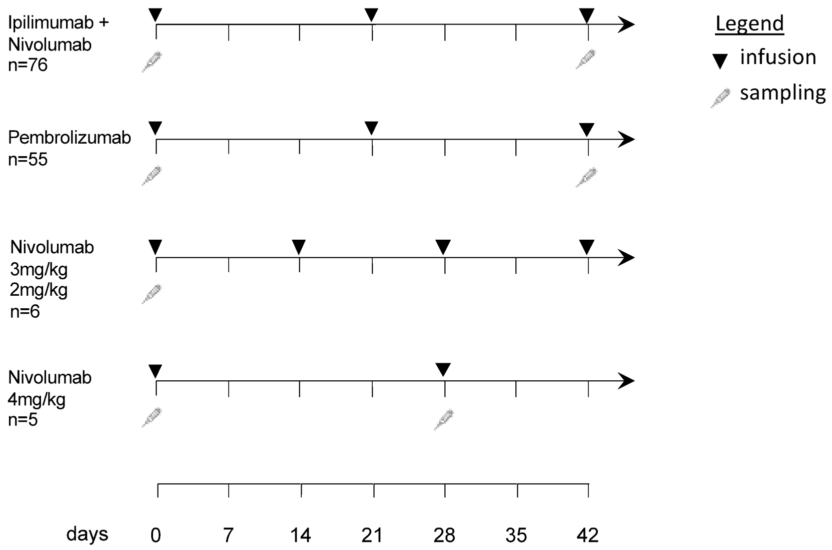

Supplementary Figure 4: Overview of the treatment and sample collection protocol.

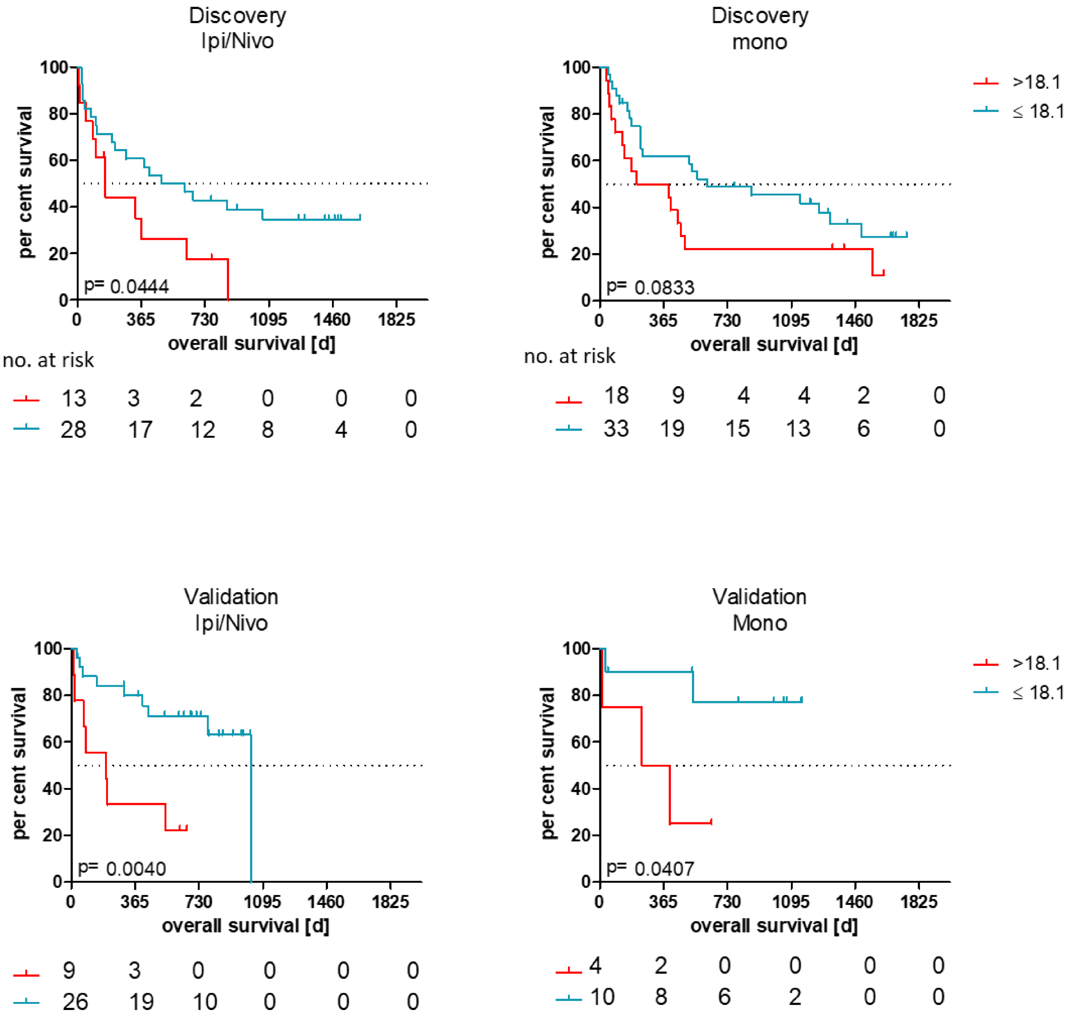

Supplementary Figure 5: Investigation of potential correlations of PD-1 mono (Mono)- versus CTLA-4 combination therapy (Ipi/Nivo) in the discovery (upper panel) and validation cohort (lower panel) with the probability of overall survival using long rank testing.

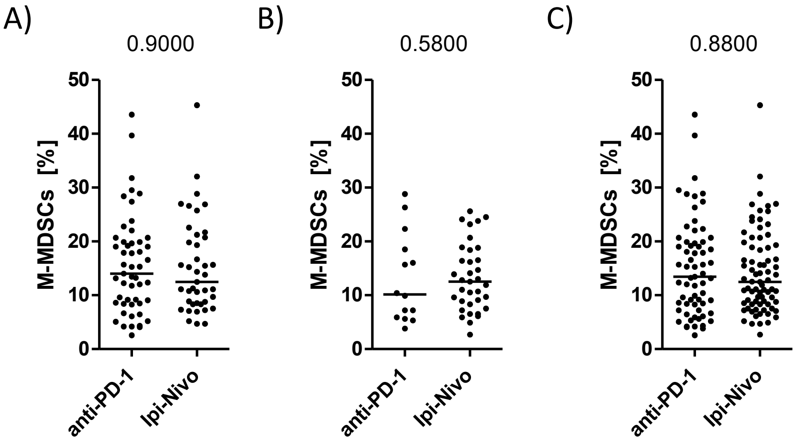

Supplementary Figure 6: Comparison of M-MDSC frequencies between PD-1 mono- (anti-PD-1) and CTLA-4 combination (Ipi-Nivo) therapy in A) the discovery, B) the validation, and C) the combined cohorts, applying Mann-Whitney-U testing. Each dot represents an individual patient and lines indicate the populations median.

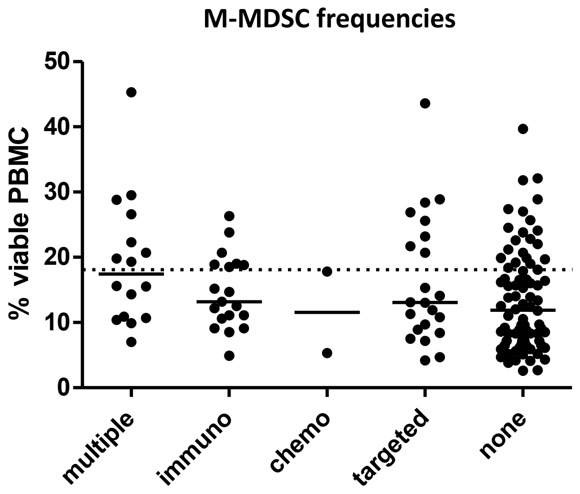

Supplementary Figure 7: Stratification of the M-MDSC frequencies of the patients of both cohorts after previous therapies. Each dot represents the M-MDSC frequency of a single patient and lines indicate the population median. Statistical evaluation using one-way ANOVA testing revealed no significant differences.

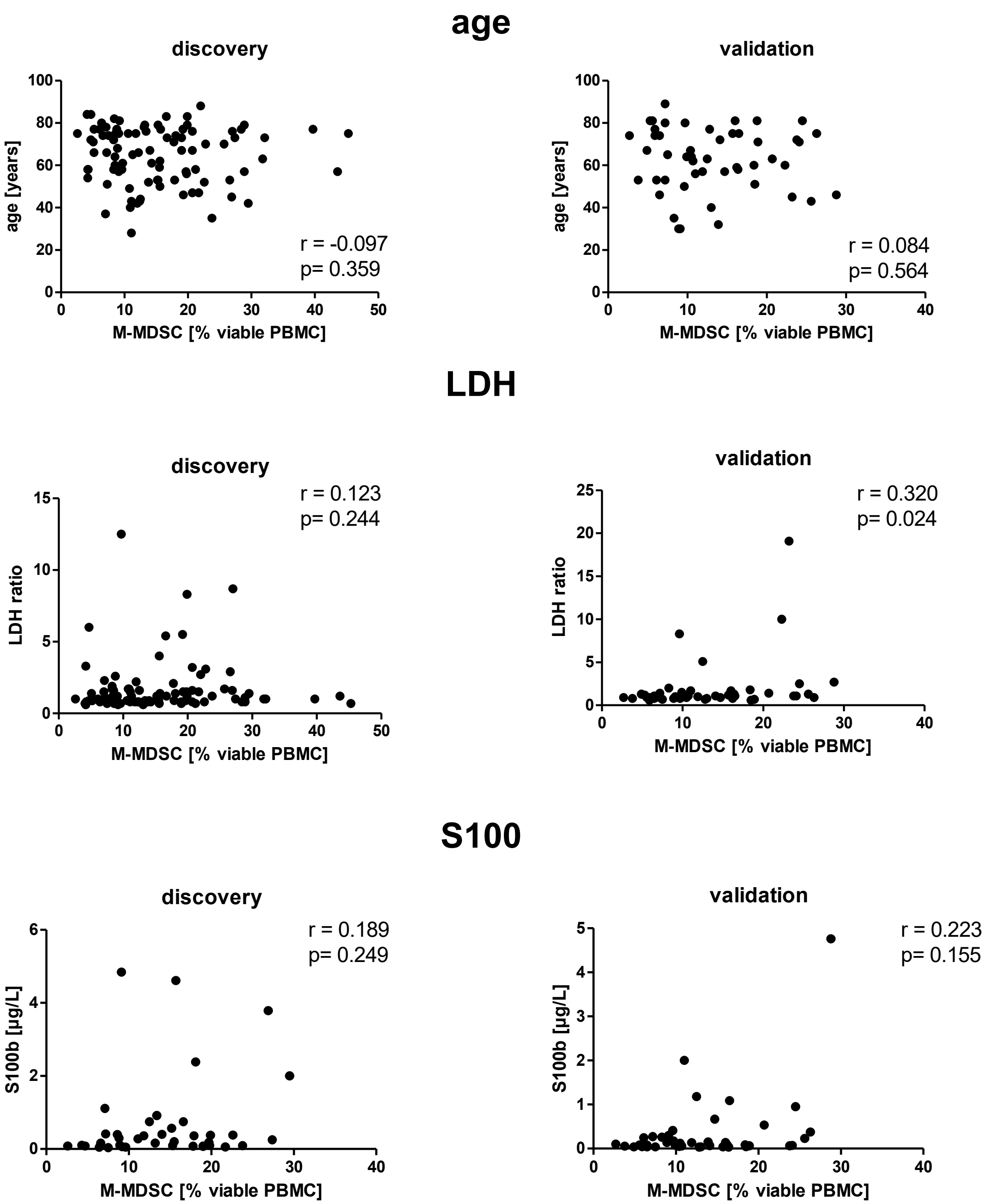

Supplementary Figure 8: Correlations of potentially confounding features of the M-MDSC frequencies in the discovery and validation cohort. Spearman R testing was performed to statistically evaluate potential correlations.

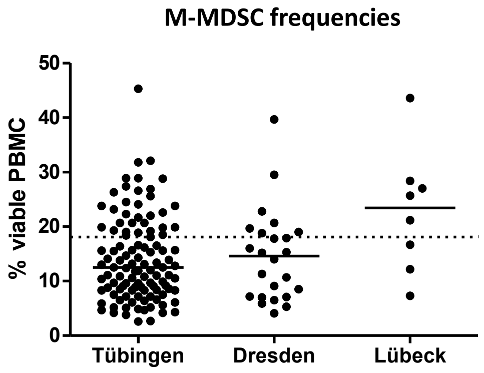

Supplementary Figure 9: Comparison of M-MDSC frequencies between the different centers. Each dot represents one M-MDSC frequency of an individual patient before start of therapy and lines indicate the populations median. One-way ANOVA testing did not reveal significant differences between the three centers.

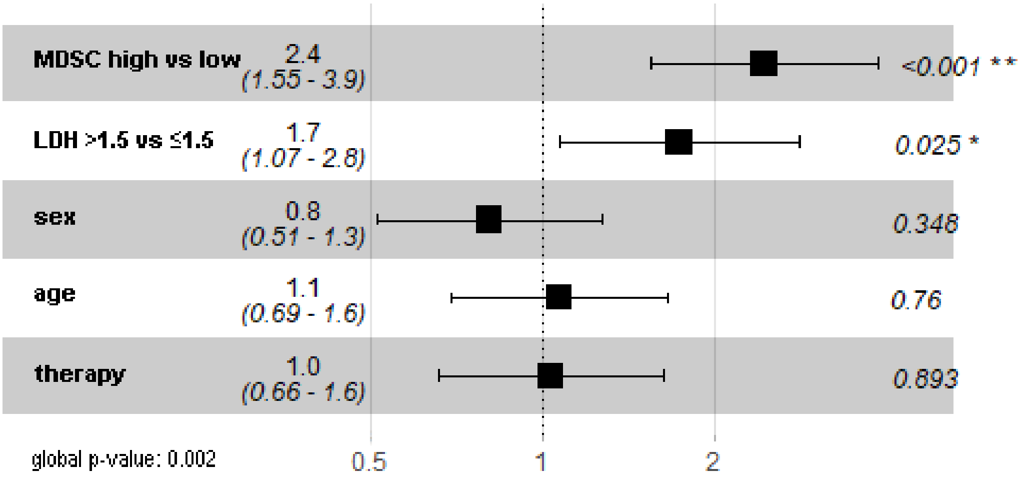

Supplementary Figure 10: Forest Plot summarizing the results of a Cox regression analysis of potential confounding features for the identified predictive characteristic of M-MDSC frequencies before the start of therapy and survival under therapy. Only high M-MDSC frequencies and LDH values correlate independently with OS. PD-1 ± CTLA-4 inhibition was used as separator in the variable “therapy”.

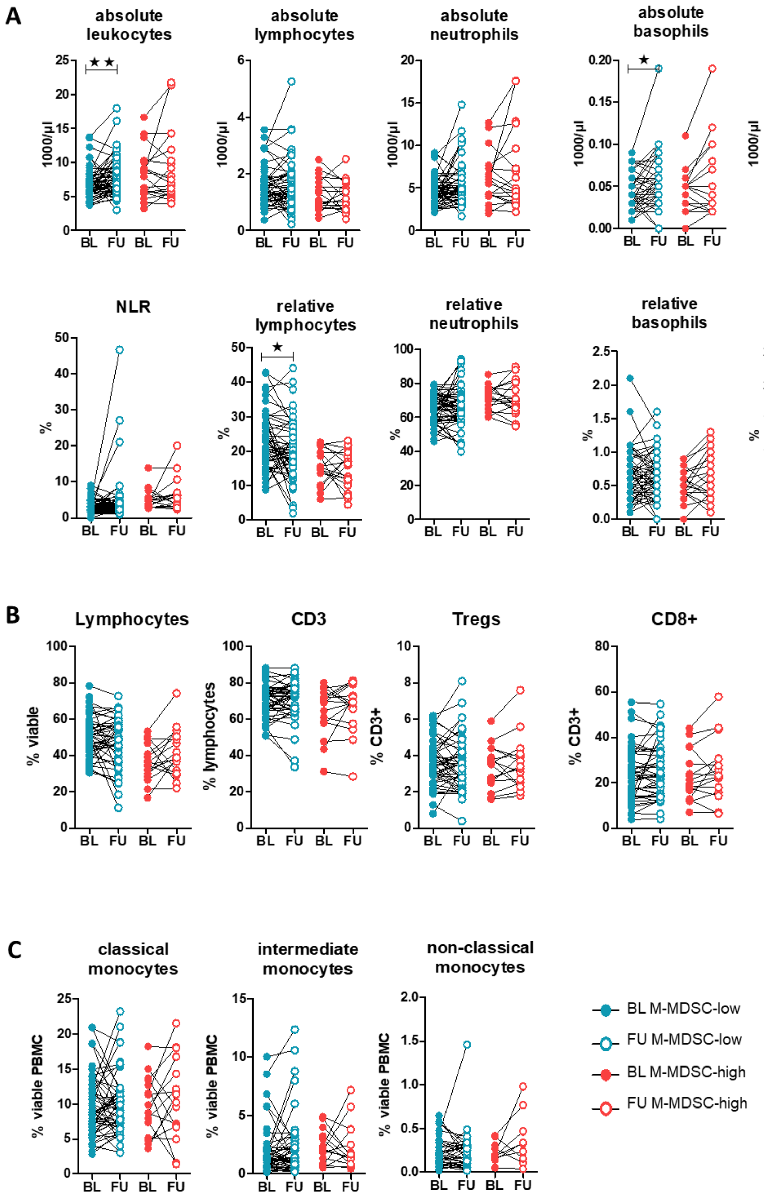

Supplementary Figure 11: Comparison of changes in the abundance of immune cell subsets under immune checkpoint blockade in M-MDSC-high versus M-MDSC-low patients: blood counts (A), lymphocytes (B) and of total myeloid cell frequencies (C).
★p<0.05 ★★p<0.01

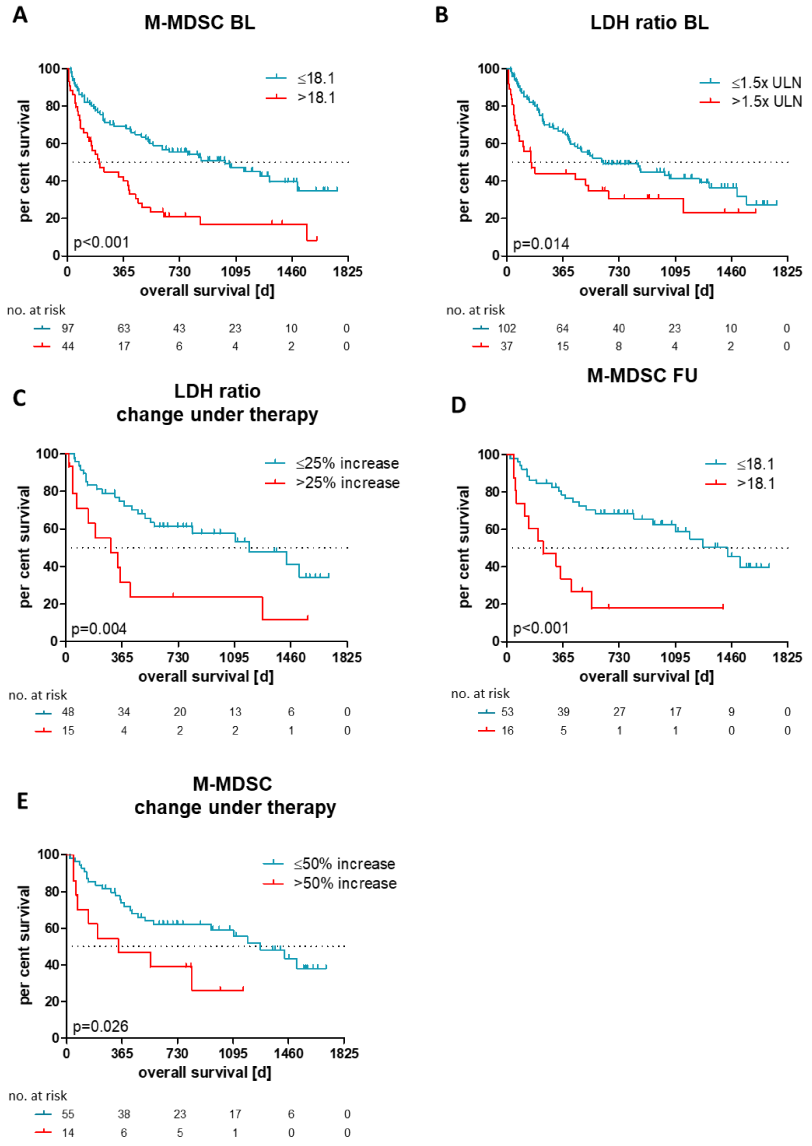

Supplementary Figure 12: Univariate analysis of the combined cohort (discovery + validation) at BL of M-MDSC frequency (A: p<0.001), LDH-ratio at BL (LDH serum level divided by the ULN (250 U/L)) (B: p=0.014) and a general increase >25% of the LDH-ratio under immune checkpoint blockade (C: p=0.004). M-MDSC frequencies at FU (D: p<0.001) and an increase of > 50% M-MDSC frequencies under immune checkpoint blockade (E: p=0.026).

BL = baseline, FU = follow up, LDH = lactate dehydrogenase, ULN= upper limit of normal

Supplementary Tables

Supplementary Table 1: Antibodies used in the myeloid cell and the T cell panel

panel	*marker*	*clone*	*fluorophore*	*vendor*	*cat*
myeloid cells	CD3	OKT3	BV605	Biolegend	317322
	CD19	HIB19	BV605	Biolegend	302244
	CD16	3G8	PacificBlue	Biolegend	302032
	CD11b	ICRF44	APC-fire	Biolegend	301352
	CD14	M5E2	PE-Cy7	Biolegend	301814
	CD33	P67.6	FITC	Biolegend	366620
	HLA-DR	L243	PerCP-Cy5.5	BD	339216
	CD56	HCD56	BV711	Biolegend	318336
	CD56	HCD56	BV605	Biolegend	318334
T cells	CD25	M-A251	PE	BD	555432
	CD127	AO19D5	BV510	Biolegend	351322
	CD8	SK1	APC-fire	Biolegend	344746
	CD8	SK1	APC-Cy7	Biolegend	344714
	CD3	UCHT1	A700	Biolegend	300424
	CD4	SK3	PerCP	BD	345770
	LAG-3	11C3C65	BV421	Biolegend	369314
	PD-1	EH12.2H7	BV711	Biolegend	329928
	TIM-3	7D3	BB515	BD	565568
	FoxP3	259DC7	A647	BD	560045
	Isotype	MOPC-21	BV421	Biolegend	400158
	Isotype	MOPC-22	BV711	Biolegend	400168
	Isotype	X40	BB515	BD	564416

Supplementary Table 2: Summary of Bonferroni corrected p-values from log rank testing of univariate overall survival correlations with blood counts.

			discovery cohort n=92		validation cohort n=49	
**factor**	**description**	**category**	**log rank p-value**	**Inter-pretation**	**log rank p-value**	**inter-pretation**
leukocytes [1000/µL]	median	≤ 7.05 vs > 7.05	0.867	failed		
	lower cutoff	< 6.11 vs ≥ 6.11	2.661	failed		
	upper cutoff	≤ 7.91 vs > 7.91	0.927	failed		
erythrocytes [1mio/µL]	Median	≤ 4.50 vs > 4.50	0.563	failed		
	lower cutoff	< 4.26 vs ≥ 4.26	0.135	failed		
	upper cutoff	≤ 4.75 vs > 4.75	2.145	failed		
thrombocytes [1000/µL]	Median	≤ 268.50 vs > 268.50	0.797	failed		
	lower cutoff	< 239.00 vs ≥ 239.00	1.926	failed		
	upper cutoff	≤ 296.00 vs > 296.00	2.787	failed		
abs. neutrophiles [1000/µL]	Median	≤ 4.66 vs > 4.66	2.268	failed		
	lower cutoff	< 4.20 vs ≥ 4.20	1.815	failed		
	upper cutoff	≤ 6.3 vs > 6.3	0.333	failed		
rel. neutros [%]	Median	≤ 70.30 vs > 70.30	2.827	failed		
	lower cutoff	< 66.00 vs ≥ 66.00	2.067	failed		
	upper cutoff	≤ 73.60 vs > 73.60	2.772	failed		
abs. eosinophile [1000/µL]	Median	≤ 0.10 vs > 1.10	2.710	failed		
	lower cutoff	< 0.11 vs ≥ 0.11	2.709	failed		
	upper cutoff	≤ 0.22 vs > 0.22	0.051	failed		
rel. eosinophile [%]	Median	≤ 1.50 vs > 1.50	1.469	failed		
	lower cutoff	< 1.40 vs ≥ 1.40	0.576	failed		
	upper cutoff	≤ 2.70 vs > 2.70	0.882	failed		
abs. basophile [1000/µL]	Median	≤ 0.04 vs > 0.04	2.290	failed		
	lower cutoff	< 0.04 vs ≥ 0.04	2.349	failed		
	upper cutoff	≤ 0.07 vs > 0.07	<0.001	candidate	0.627	failed
rel. basophile [%]	Median	≤ 0.50 vs > 0.50	1.805	failed		
	lower cutoff	< 0.50 vs ≥ 0.50	1.736	failed		
	upper cutoff	≤ 0.80 vs > 0.80	0.513	failed		
abs. monocytes [1000/µL]	Median	≤ 0.61 vs > 0.61	0.123	failed		
	lower cutoff	< 0.52 vs ≥ 0.52	0.549	failed		
	upper cutoff	≤ 0.76 vs > 0.76	0.051	failed		
rel. monocytes [%]	Median	≤ 8.55 vs > 8.55	1.724	failed		
	lower cutoff	< 8.00 vs ≥ 8.00	2.085	failed		
	upper cutoff	≤ 10.00 vs > 10.00	0.285	failed		
abs. lymphozyten [1000/µL]	Median	≤ 1.32 vs > 1.32	1.649	failed		
	lower cutoff	< 1.07 vs ≥ 1.07	1.095	failed		
	upper cutoff	≤ 1.69 vs > 1.69	2.190	failed		
rel. lymphozyten [%]	Median	≤ 18.05 vs > 18.05	1.555	failed		
	lower cutoff	< 15.40 vs ≥ 15.40	2.019	failed		
	upper cutoff	≤ 22.70 vs > 22.70	1.086	failed		
NLR	Median	≤ 3.80 vs > 3.80	1.119	failed		
	lower cutoff	< 3.20 vs ≥ 3.20	1.104	failed		
	upper cutoff	≤ 5.10 vs > 5.10	2.655	failed		

Supplementary Table 3: Summary of Bonferroni corrected p-values from log rank testing of univariate overall survival correlations with T cell populations assed by flow cytometry.

			discovery cohort n=92		validation cohort n=49	
**factor**	**description**	**category**	**log rank p-value**	**inter-pretation**	**log rank p-value**	**inter-pretation**
Lymphocytes	median	≤ 50.6 vs > 50.6	0.771	failed		
	lower cutoff	< 42.3 vs ≥ 42.3	2.661	failed		
	upper cutoff	≤ 53.4 vs > 53.4	2.286	failed		
CD3 [%Lymphocytes]	Median	≤ 68.5 vs > 68.5	2.260	failed		
	lower cutoff	< 61.2 vs ≥ 61.2	2.994	failed		
	upper cutoff	≤ 74.9 vs > 74.9	0.141	failed		
Tregs [%CD3]	median	≤ 3.6 vs > 3.6	0.491	failed		
	lower cutoff	< 3.1 vs ≥ 3.1	0.129	failed		
	upper cutoff	≤ 74.9 vs > 74.9	0.306	failed		
LAG3 [%Tregs]	median	≤ 1.4 vs > 1.4	2.438	failed		
	lower cutoff	< 1.2 vs ≥ 1.2	2.010	failed		
	upper cutoff	≤ 2 vs > 2	2.406	failed		
PD1 [%Tregs]	median	≤ 15.8 vs > 15.8	1.733	failed		
	lower cutoff	< 13.5 vs ≥ 13.5	2.091	failed		
	upper cutoff	≤ 17.9 vs > 17.9	1.308	failed		
TIM3 [%Tregs]	median	≤ 10.9 vs > 10.9	2.852	failed		
	lower cutoff	< 9 vs ≥ 9	1.371	failed		
	upper cutoff	≤ 13.4 vs > 13.4	0.396	failed		
CD4 without Tregs [%CD3]	median	≤ 61 vs > 61	1.785	failed		
	lower cutoff	< 54.1 vs ≥ 54.1	1.677	failed		
	upper cutoff	≤ 66.5 vs > 66.5	2.595	failed		
LAG3 [%CD4 without Tregs]	median	≤0.3 vs > 0.3	2.669	failed		
	lower cutoff	< 0.4 vs ≥ 0.4	2.670	failed		
	upper cutoff	≤ 0.9 vs > 0.9	0.231	failed		
PD1[%CD4 without Tregs]	median	≤ 11.1 vs > 11.1	1.516	failed		
	lower cutoff	< 8.6 vs ≥ 8.6	0.903	failed		
	upper cutoff	≤ 14.7 vs > 14.7	2.637	failed		
TIM3[%CD4 without Tregs]	median	≤ 5.7 vs > 5.7	0.763	failed		
	lower cutoff	< 4.6 vs ≥ 4.6	2.820	failed		
	upper cutoff	≤ 7.8 vs > 7.8	2.307	failed		
CD8 [%CD3]	median	≤ 20.0 vs > 20.0	2.806	failed		
	lower cutoff	< 16.9 vs ≥ 16.9	2.013	failed		
	upper cutoff	≤ 25.2 vs > 25.2	2.454	failed		
CD25 [%CD8]	median	≤ 7.0 vs > 7.0	2.955	failed		
	lower cutoff	< 5.6 vs ≥ 5.6	2.451	failed		
	upper cutoff	≤ 12.9 vs > 12.9	1.974	failed		
LAG3 [%CD8]	median	≤ 0.5 vs > 0.5	2.108	failed		
	lower cutoff	< 0.9 vs ≥ 0.9	0.561	failed		
	upper cutoff	≤ 1.7 vs > 1.7	1.545	failed		
PD1 [%CD8]	median	≤ 18.1 vs > 18.1	1.721	failed		
	lower cutoff	< 13.9 vs ≥ 13.9	2.898	failed		
	upper cutoff	≤ 25.9 vs > 25.9	0.657	failed		
TIM3 [%CD8]	median	≤ 14.8 vs > 14.8	0.869	failed		
	lower cutoff	< 11.9 vs ≥ 11.9	1.263	failed		
	upper cutoff	≤ 21.0 vs > 21.0	2.154	failed		

Supplementary Table 4: Summary of Bonferroni corrected p-values from log rank testing of univariate overall survival correlations with myeloid cell populations assed by flow cytometry.

			discovery cohort n=92		validation cohort n=49	
factor	description	category	log rank p-value	inter-pretation	log rank p-value	inter-pretation
M-MDSC	median	≤ 13.8 vs > 13.8	2.179	failed		
	lower cutoff	< 10.8 vs ≥ 10.8	1.211	failed		
	**upper cutoff**	**≤ 18.1 vs > 18.1**	**0.030**	**candidate**	**<0.001**	**confirmed**
classical monocytes	median	≤ 8.7 vs > 8.7	1.264	failed		
	lower cutoff	< 6.6 vs ≥ 6.6	0.719	failed		
	upper cutoff	≤ 10.8 vs > 10.8	1.925	failed		
intermediate monocytes	median	≤ 1.2 vs > 1.2	2.031	failed		
	lower cutoff	< 1.4 vs ≥ 1.4	1.853	failed		
	upper cutoff	≤ 3.0 vs > 3.0	2.327	failed		
non-classical monocytes	median	≤ 0.2 vs > 0.2	2.081	failed		
	lower cutoff	< 0.2 vs ≥ 0.2	0.819	failed		
	upper cutoff	≤ 3.0 vs > 3.0	0.179	failed		

Supplementary Table 5: Summary of median frequencies, counts, interquartile range (IQR) of the investigated cell populations in patients in the M-MDSC-low versus M-MDSC-high group. P-values have been determined using Mann-Whitney U testing.

	cell population	p	M-MDSC low median	M-MDSC low IQR	M-MDSC high median	M-MDSC high IQR
serum blood parameters	leukocytes [1000/µL]	**0.015**	6.8	2.7	8.2	4.0
	erythrocytes[1mio/µL]	0.817	4.5	0.7	4.5	0.8
	thrombocytes [1000/µL]	0.161	252	85.3	280.5	79.8
	abs. neutrophils [1000/µL]	**0.001**	4.4	2.5	6.0	3
	rel. neutrophils [%]	**<0.001**	66	12.3	75	9.5
	abs. eosinophils [1000/µL]	**0.044**	0.1	0.1	0.1	0.1
	rel. eosinophils [%]	**0.005**	1.9	1.7	1	1.4
	abs. basophils [1000/µL]	0.973	<0.1	<0.1	<0.1	0.1
	rel. basophils [%]	0.127	0.6	0.4	0.4	0.4
	abs. monocytes [1000/µL]	**0.001**	0.6	0.3	0.7	0.3
	rel. monocytes [%]	0.131	8.5	2.6	9	2.6
	abs. lymphocytes [1000/µL]	**<0.001**	1.5	0.8	1.0	0.7
	rel. lymphocytes [%]	**<0.001**	21.8	11.1	14.4	6.5
	NLR	**<0.001**	3.0	2.2	5.1	4.1
flow cytometry	lymphocytes	**<0.001**	51.4	17.5	32.6	13.5
	CD3+ [lymphocytes]	**0.015**	72	10.6	68.0	18.4
	Tregs [CD3]	0.378	3.8	1.7	4.0	1.9
	LAG-3+Tregs	0.981	1.1	1.3	1.2	1.3
	PD-1+Tregs	**0.025**	12.5	9.7	14.1	7.1
	TIM-3+Tregs	0.761	8.5	7.5	8.7	6.7
	CD4+ [CD3]	0.338	61	17.6	60.2	21
	LAG-3+CD4	0.554	0.3	0.3	0.3	0.4
	PD-1+CD4	0.131	10.2	8.7	10.5	9.4
	TIM-3+CD4	0.988	4.5	4.0	5.0	4.7
	CD8+ [CD3]	0.759	21.5	14.4	20.6	19.1
	CD25+CD8	0.655	7.5	13.2	6.3	7.7
	LAG-3+CD8	0.511	0.5	1.2	0.75	1.4
	PD-1+CD8	0.146	15.4	12.5	18.3	20.2
	TIM-3+CD8	0.251	10.9	12.0	11.5	15.4
	classical monocytes [viable PBMCs]	0.101	8.7	5.8	10.0	6.8
	intermediate monocytes [viable PBMC]	**0.031**	1.1	1.4	1.4	2.1
	non-classical monocytes [viable PBMC]	0.389	0.2	0.2	0.1	0.2
